# Supplementary material for: Self-compassion and association with distress, depression, and anxiety among displaced Syrians: A population-based study
Source: PLoS One. 2024 Sep 19;19(9):e0309051. doi: 10.1371/journal.pone.0309051 (PMC11412651; doi:10.1371/journal.pone.0309051)
Supplement: Attachment — Submitted filename: REVIEW NOTES.docx [file pone.0309051.s003.docx]

Comments to the Authors

Regarding the article, I have found significant strengths, especially for the findings of the study. In addition, the study may address the predictive roles of resilience and self-compassion for mental health outcomes in a disadvantaged population. However, there are some issues that may limit the potential contributions of this study. I hope that the authors will find helpful the following issues that I pointed.

**Introduction**

The introduction of the manuscript requires improvement. One of my main concern is the lack of clarity regarding the novelty of the study, what this study adds novelty in relation to the existing literature. It is recommened to emphasize the relationship between resilience, self-compassion, and mental health outcomes, rather than simply saying that no study has examined these variables among refugees-this is not sufficient to establish novelty-. Another concern is the limited knowledge about the related variables and functions for traumatized individuals. Please, include the existing literature to understand the association of these variables in the scope of mental health. Lastly, please clarify the aim of the study.

Line.73 Please explain what you mean “work upstream”

Line.80-82. Self-compassion has been worked with refugees in contemporary research. Please check the relevant sentence and the related articles Rashidian et al. (2023), Ghasemy (2020) etc

Line.82-83. *“Furthermore, the link between self-compassion and resilience is explored in the literature, but not defined in the context of displaced populations”*

How these two factors, self-compassion and resilience, are associated in other sample as you stated in this sentence? Please, add more information.

**Materials and Methods**

Consider reorganizing this section by adding subtitles under methods and materials, such as participants, procedure, statistical analysis etc. Please refer to the journal archieves for guidance on how to organize this part.

Did you have any inclusion or exclusion criteria for sample selection in your study? If you had, please indicate.

Line.102-105. You excluded one participants as being younger than 18 years old. However, when I examined the Table 1, I noticed that the age section had a “not reported” category. How did you determine that these 10 participants were older than 18 or younger than 80?

Line. 102-105. How did you detect social desirability in your sample? Did you use any validated measurement tool for identifying it?

Line. 106-109. I assume what you mean by “the quality scores” is missing values in dataset. If not, could you explain how you determined the quality of the data. I suggest that you provide statistical evidence why you excluded these data.

Measurement tools:

You employed Arabic version of all the scales. I wonder about the psychometric properties of these scales. Do the scales have adequate validity and realibility scores for Arabic individuals? In addition, how about the internal consistency scores of your sample for all measurement tools?

Line.118-128. Indeed, the HSCL-25 has two sub-dimensions: anxiety and depression. According to the references you cite (18 and 19) researchers have also calculated anxiety and depression seperately. The Arabic version (Fares et al., 2021) recommended bifactor model: two factors (anxiety/depression) and a thir factor (underlying 25-items). However, you calculated a total score for emotional distress and 15 items for depression separately- it was not calculated any score for anxiety-.The introduction part mentioned that anxiety is also common adverse mental health outcomes among refugees. I wonder why you did not measure anxiety for your sample?

I assume you have used the information for HSCL-25 and calculation of its from this website <https://hprt-cambridge.org/screening/hopkins-symptom-checklist>. I recommend that you cite this website in your manuscript or other articles which point the same total score and depression sub-dimension. Additionally, please paraphrase the sentences, as there is a high degree of similarity between your sentences and the text on this website.

Line.133 “This will be a secondary exposure to self-compassion measures” I did not understand what you meant with this sentence. Please explain.

Line.144-151. To make this section easier to read and avoid confusion, I suggest titling it as “statistical analysis”

This section requires additional information regarding the statistics that the authors used. Were there any missing values? As I read previous paragraph that you wrote (for participation selection), some individuals did not complete all items in the data set. Thus, please include information about missing value. If any missing values, how were missing values handled. There is some assumptions for conducting ANOVA. I wonder whether these assumptions were checked or not. Specifically, was the normality of the data checked? Did data distribute normally? Please check the assumptions of ANOVA and explain it in a clear way in the text.

Line.144.Which version of SPSS was used? All the statistics that you mentioned could be carried out in SPSS? Why did you use excel?

Line.144-146. How were the low, moderate, and high self-compassion scores of the participants determined? I knew that this scale does not have any cut-off scores. (If wrong, please inform me and add the text)

Line.146 “…. ANOVA followed by post hoc Tukey’s Honest Significance Test with Bonferroni correction”. Did you use Tukey or Bonferroni? I am confused.

Line.148. Please rewrite the sentence in a clearer way. For instance, we used univariate logistic regression for ….. and multivariate for ….. etc.

Results

In general, results section is a kind of complicated. It is hard to understand and follow the findings. I advise the authors adding subtitle under results section to make easier the interpretation for instance descriptive analysis, prevalence, logistic regression analysis -for emotional distress, for depression etc. This will be helping to increase understandability.

Line.174-176. Please, explain why did you conduct correlation? If you have a hypothesis, please indicate in the text or you may think to exclude it.

Line.203-211. As I suggested above, maybe you can add distinct subheadings for emotional distress and depression.

Discussion

In general, it is recommended that the authors may present their finding at first, and then discuss them based on the existing literature, providing possible explanations of their findings. Secondly, the authors discussed only findings of resilience and self-compassion. However, gender, meeting financial needs, and TEC were also found to be significant predictors of emotional distress and depression in logistic regression. Please, include a discussion about these findings in this part. Lastly, what does this study suggest for future studies or clinical implications? Please, provide your suggestions in a separate paragraph.

Please indicate that Jordan is one of the LMICs according to (World Bank?) …. Since you interpreted migration to LMICs could be an explanation.

Line.241 The authors specifically emphasized the term “severely traumatized…” in some sentences. When you said that, I expected an objective indicator that provides the trauma level of your sample or your sample just consisted of individuals with high levels of traumatic stress. I believe that simply stating this term will not enough to prove all of the sample experienced severe trauma. Maybe instead of emphasizing the term so much, it may be sufficient to briefly introduce about the trauma that experienced by Syrian refugees in the introduction part.

Line.249-250. As I stated previously, there have been some studies conducted on self-compassion among refugees. Please check it and add.

“***Greater self-compassion is associated with less mental health morbidity***”

First, you can present your findings of this study. Then, you can discuss how your finding placed in the existing literature. The psychosocial intervention findings that you cited may be incorporated into clinical or future implications by combining them with your findings.

**“Self-compassion is a consistent predictor of lower emotional distress and depression independent of resilience”**

Please explain the finding of why resilience did not predict emotional distress and depression based on existing literature. You cited an article numbered 6, how they explain this finding? Moreover, some researchers argued that being resilient may not be associated with a reduction in distress (e.g., Blanke et al., 2023).

Line.274-283. The authors discussed this correlation finding. However, as previously stated, you should determine the aim of the manuscript and if you wish to include this finding, please clarify the aim of the study in introduction part.
