# Supplementary material for: Self-compassion and association with distress, depression, and anxiety among displaced Syrians: A population-based study
Source: PLoS One. 2024 Sep 19;19(9):e0309051. doi: 10.1371/journal.pone.0309051 (PMC11412651; doi:10.1371/journal.pone.0309051)
Supplement: Attachment — Submitted filename: Plos_One Revised Version Review.docx [file pone.0309051.s005.docx]

**Self-compassion and association with distress, depression, and anxiety among displaced Syrians: a population-based study**

Regarding the revision of the manuscript, I believe that this version exhibits greater strength than the previous version. I would like to appreciate the authors for their efforts.

**In the abstract**

In the abstract, depending on your findings, you might consider adding the predictive role of gender, financial status and traumatic exposure The incorporation of these variables, you might think about making abstract more attractive and interesting.

**In Methods and Results sections**

Lines 198-199. In the study population section, you wrote: “In total, 47 surveys with a quality score of 2 or 3 were excluded from the final analysis.”. However, in this sentence you wrote “Missing values were conservatively given a value of zero and included in the analysis”. As before, you stated you exclude the data with missing values. Please, check it.

You applied non parametric test with the inclusion of anxiety. Regarding this information, I understand that the anxiety measurement was not normally distributed but what about other measurement such as depression, emotional distress? If only anxiety was non-normally distributed, I recommend using Kruskal-Wallis H test for anxiety, one-way ANOVA for other measurements if normal distrubiton was met.
