# Supplementary material for: Self-compassion and association with distress, depression, and anxiety among displaced Syrians: A population-based study
Source: PLoS One. 2024 Sep 19;19(9):e0309051. doi: 10.1371/journal.pone.0309051 (PMC11412651; doi:10.1371/journal.pone.0309051)
Supplement: Attachment — Submitted filename: Response to Reviewers_7.21.docx [file pone.0309051.s006.docx]

**Self-compassion and association with distress, depression, and anxiety among displaced Syrians: a population-based study**

-Regarding the revision of the manuscript, I believe that this version exhibits greater strength than the previous version. I would like to appreciate the authors for their efforts.

Response:

*Thank you for the all thoughtful feedback you have provided which has ultimately strengthened this paper.*

**In the abstract**

-In the abstract, depending on your findings, you might consider adding the predictive role of gender, financial status and traumatic exposure The incorporation of these variables, you might think about making abstract more attractive and interesting.

Response:

*We included this finding in the abstract. Thank you for this suggestion.*

**In Methods and Results sections**

-Lines 198-199. In the study population section, you wrote: “In total, 47 surveys with a quality score of 2 or 3 were excluded from the final analysis.”. However, in this sentence you wrote “Missing values were conservatively given a value of zero and included in the analysis”. As before, you stated you exclude the data with missing values. Please, check it.

Response:

*Thank you for bringing this to our attention. We edited the text to further clarify the quality score assigned to each survery. To summarize, surveys received a quality score of if they had minimal missing data, 2 if they were missing more than 50% of the data in one section, and 3 if they were missing data in multiple sections. Missing values from surveys with a quality score of 1 were then conservatively given a value of zero.*

-You applied non parametric test with the inclusion of anxiety. Regarding this information, I understand that the anxiety measurement was not normally distributed but what about other measurement such as depression, emotional distress? If only anxiety was non-normally distributed, I recommend using Kruskal-Wallis H test for anxiety, one-way ANOVA for other measurements if normal distrubiton was met.

Response:

*Thank you for this comment. Depression, emotional distress, and anxiety were not normally distrubted across self compassion groups which is why Kruskal-Wallis H test was ultimatley applied. Absence of normality was determined by a Shapiro-Wilk test with p < 0.05.*

Editor Comments:

-Abstract: Please simplify the abstract removing the statistical results such as p values and OR, and focusing on the summary of your research findings and contributions (please also see the reviewer's comment on the abstract)

Response:

*We removed the statistical results from the abstract and included more of our findings per the reviewers suggestion (ie gender, financial status, and trauma exposure).*

-I suggest avoiding terms like “mental illnesses” or “mental health disorders” (for instance see the first paragraph in the introduction, lines 59-60, or page 5., lines 104 and 111; please check it throughout the manuscript), as they might refer clinical settings and not appropriate for non-hospitalised vulnerable groups like refugees. You could adopt the terms such as mental health problems instead.

Response:

*Thank you for this suggestion. The terms “mental illnesses” and “mental health disorders” have been removed from the manuscript and replaced with “poor mental health” or “mental health problems”.*
